# Effectiveness of Hearing Aid Adherence on Social Connectivity in Older Adults: A Scoping Review

**DOI:** 10.1093/geroni/igaf122.2780

**Published:** 2025-12-31

**Authors:** Fumiko Hamada, Charity Lewis, Lindsay Peterson

**Affiliations:** University of South Florida School of Aging, Tampa, Florida, United States; University of South Florida, Tampa, Florida, United States; University of South Florida, Tampa, Florida, United States

## Abstract

Age-related hearing loss affects 65% of older adults but is widely untreated. Untreated hearing loss is associated with multiple adverse outcomes, including social isolation and loneliness. This scoping review aims to assess the effectiveness of hearing aid use on social connectivity in adults ages 50 and older living with age-related hearing loss, and the review was performed using PubMed, PsycINFO, and Web of Science databases. Search terms included older adult, hearing aid, age-related hearing loss, social connectivity, social engagement, social isolation, and loneliness. English-language studies with participants aged over 50 years diagnosed with age-related hearing loss treated with the use of hearing aids were included. A total of 375 articles were identified, of which six met the inclusion criteria. Several outcome measures were used: Three studies focused on social isolation, loneliness, or both. One study investigated the relationship between social isolation and hearing loss and cognition, while another examined the impact of hearing aid use on social participation. All studies examining social isolation and loneliness reported improved scores following the adoption of hearing aids. One study showed that hearing aid use mediated the link between hearing loss and cognitive decline, while another found that increased usage (wearing them for more hours) improved social participation. This scoping review concluded that hearing aid adherence may help to strengthen social connectivity among older adults. Given that hearing aid use is an effective treatment for age-related hearing loss, there is a substantiated need for policy and governmental involvement to improve access and affordability.

